# Retinopathy of prematurity as a clinical marker of developmental vulnerability in preschool children: A nationwide Korean Health Screening study

**DOI:** 10.1371/journal.pone.0356773

**Published:** 2026-09-02

**Authors:** Tae-Eun Kim, Hye Won Park, Sanghyun Park, Hyun Jin Shin

**Affiliations:** 1 Department of Clinical Pharmacology, Konkuk University Medical Center, Seoul, Republic of Korea; 2 Department of Pediatrics, Konkuk University Medical Center, Konkuk University School of Medicine, Seoul, Republic of Korea; 3 Department of Ophthalmology, Konkuk University Medical Center, Konkuk University School of Medicine, Seoul, Republic of Korea; 4 Research Institute of Medical Science, Konkuk University School of Medicine, Seoul, Republic of Korea; Saitama Medical Center: Saitama Ika Daigaku Sogo Iryo Center, JAPAN

## Abstract

**Purpose:**

To determine the Retinopathy of prematurity (ROP) and its association with developmental delay in Korean preschool children.

**Methods:**

We conducted a nationwide cohort study using the Korean National Health Insurance Service and National Health Screening Program for Infants and Children (NHSPIC). The study included 808,536 children born 2014–2018 who completed the 7th NHSPIC screening at 54–60 months. Development assessed by the Korean Developmental Screening Test (K-DST) in six domains (self-care, cognition, language, fine motor, gross motor, and sociality. Subnormal visual acuity (VA) was defined as <0.6 (or 0.63) in either eye. Multivariable logistic regression estimated adjusted odds ratios (ORs) for developmental delay.

**Results:**

Of 808,536 children, 7,428 (0.92%) had a history of ROP. Children with ROP had lower birth weight and gestational age and higher frequencies of maternal complications and subnormal presenting VA. After adjustment for available maternal and child covariates, ROP was associated with higher odds of developmental delay across several domains, with adjusted ORs ranging from 1.35 for cognition (95% CI: 1.00–1.81) to 1.56 for gross motor development (95% CI: 1.18–2.07). In analyses stratified by ROP and presenting VA status, children.

**Conclusions:**

Children with a history of ROP showed higher odds of developmental delay at 54–60 months, especially when subnormal presenting visual acuity was also present. ROP may serve as a clinically useful marker of prematurity-related developmental vulnerability, supporting integrated ophthalmic and developmental surveillance before school entry.

## Introduction

Retinopathy of prematurity (ROP) is a vasoproliferative ocular condition that can occur in infants who are premature (born at <36 weeks of gestation) or who weigh <1.5 kg at birth (low-birth-weight infants). ROP primarily affects premature infants and is recognized as a leading cause of childhood blindness worldwide [1,2]. While significant advancements in early diagnosis and treatment such as timely screening and management have demonstrably reduced the incidence of ROP-induced permanent blindness, the overall prevalence of ROP has (paradoxically) increased globally [3,4]. This increase is largely attributed to improved neonatal care and obstetric medicine, which have increased the survival rates of preterm infants across low-income and middle-income countries [5,6].

Although ROP has been extensively studied in terms of classification, treatment, and visual prognosis, its relationship with broader developmental outcomes remains incompletely understood. This relationship is difficult to interpret because ROP is strongly associated with prematurity, low birth weight, neonatal illness severity, oxygen exposure, and other perinatal factors that also influence neurodevelopment. Recent evidence suggests that ROP may represent one clinical manifestation of broader neurovascular vulnerability in preterm infants rather than an isolated ocular disease [7,8]. Previous studies have linked ROP to cognitive impairment, intellectual disability, cerebral palsy, and behavioral problems [9–13], but the relative contributions of ROP, visual impairment, and shared prematurity-related factors remain uncertain. Therefore, population-based characterization of developmental screening outcomes in children with a history of ROP may help identify children who warrant closer ophthalmic and developmental follow-up.

South Korean government operates a robust national system that monitors child health and development. The National Health Screening Program for Infants and Children (NHSPIC) is a nationwide initiative that provides comprehensive health assessments at eight distinct intervals from 1 to 71 months of age. This program integrates evaluations of visual acuity (VA), physical growth, and developmental status utilizing the Korean Developmental Screening Test (K-DST), which is a validated, caregiver-completed questionnaire designed to assess six core developmental domains: sociality (from 9 months onward), and self-care, cognition, language, fine motor skills, and gross motor skills (from 18 months onward). This extensive national screening infrastructure provides a unique and invaluable opportunity to investigate the long-term health and developmental outcomes associated with various pediatric conditions in a large and representative population.

This study analyzed the extensive national data obtained in the NHSPIC to compare overall developmental outcomes as measured using the K-DST between children with and without a diagnosis of ROP. Furthermore, we sought to understand the specific risks of developmental delay by stratifying ROP patients based on their visual function, differentiating between those with normal and subnormal VA. This investigation was designed to understand of how ROP and its associated visual function may differentially influence various aspects of early childhood development in a large, population-based cohort.

## Methods

### Data source

This study adhered to the principles of the Declaration of Helsinki and was approved by both the National Health Insurance Service (NHIS) of Korea and the Institutional Review Board of Konkuk University Medical Center (KUMC IRB 2025-07-021). The data were last accessed for research purposes on 11/09/2025. We utilized nationwide, large-scale datasets drawn from the NHIS and NHSPIC. The NHIS administers a universal, single-payer health insurance system that covers >97% of the Korean population, which means that comprehensive data are collected. The resulting integrated database includes demographic information, ICD-10 diagnostic codes, clinical treatment records, prescription data, and maternal medical histories.

The NHSPIC is a government-funded health screening initiative that provides standardized checkups free of charge to all infants and young children in South Korea. Screening assessments are conducted at eight developmental milestones between 2 weeks and 71 months of age. These assessments include anthropometric measurements, eye examinations, and developmental evaluations using the K-DST. This systematic and inclusive approach ensured both high participation rates and reliable data quality.

### Study population

We constructed a mother–offspring cohort from the NHIS database that included all live births to mothers aged 20–44 years in South Korea between January 1, 2014 and December 31, 2018, with linked maternal health records. The present analysis focused on developmental screening outcomes among children without previously coded major neurodevelopmental or neurologic diagnoses before the 7th NHSPIC screening. Therefore, children with diagnostic codes for autism spectrum disorder, speech or language developmental disorders, unspecified developmental disorders, attention-deficit/hyperactivity disorder, or delayed developmental milestones were excluded. Children with cerebral palsy, periventricular leukomalacia, neonatal cerebral ischemia, hypoxic-ischemic encephalopathy, anoxic brain damage, or major congenital brain anomalies, including anencephaly, microcephaly, and other brain malformations, were also excluded. In addition, children with chromosomal abnormalities, including Down syndrome, Edwards syndrome, Patau syndrome, 5p deletion syndrome, Angelman syndrome, Klinefelter syndrome, and Turner syndrome, were excluded. These criteria were applied to characterize developmental screening abnormalities in children without previously established major neurodevelopmental diagnoses, rather than to represent the full spectrum of neurodevelopmental impairment among children with ROP. The final analysis included children who completed the 7th NHSPIC screening at 54–60 months of age. ROP was identified using the ICD-10 diagnostic code H35.1.

### Development evaluation

Developmental outcomes were assessed using the K-DST, which is included in the NHSPIC screening stages performed at 9–12, 18–24, 30–36, 42–48, 54–60, and 66–71 months [14]. The K-DST measures six developmental domains: language, gross motor skills, fine motor skills, cognition, sociality, and self-care. Each domain contains eight items rated on the following 4-point scale: 3 (“can do well”), 2 (“can do to some extent”), 1 (“can hardly do”), and 0 (“cannot do at all”). Domain scores therefore range from 0 to 24, with higher values indicating better developmental performance. Scores are categorized relative to population norms into advanced (>+1 SD), appropriate (−1 SD to +1 SD), monitoring required (−1 to −2 SD), and further assessment indicated (<−2 SD). A score below −2 SD corresponds to roughly the lowest 2.3% of the age group and indicates a high probability of developmental delay. In this study we used K-DST measurements from the 7th NHSPIC screening stage performed at 54–60 months. Children were classified as developmentally delayed if they scored below −2 SD in any domain [15].

### VA assessments

VA was evaluated during the 6th (42–48 months), 7th (54–60 months), and 8th (66–71 months) screening stages of the NHSPIC and was recorded using the decimal visual acuity scale. This study used VA measurements from the 7th screening stage, corresponding to the same screening period as the developmental assessment. According to the NHSPIC vision screening guidelines, children are referred for further ophthalmologic evaluation if their presenting decimal VA is < 0.5 at the 5th screening stage or <0.6, or <0.63 depending on the optotype chart format, at the 6th and 7th screening stages. Presenting VA refers to visual acuity measured under habitual visual conditions, either with usual corrective lenses if worn or without correction if no correction is used. Therefore, presenting VA reflects the child’s functional visual status in daily life, although it does not distinguish the underlying cause of reduced vision or represent best-corrected VA after cycloplegic refraction. In this study, subnormal presenting VA was defined as decimal VA < 0.6, or <0.63 depending on the chart format, in at least one eye at the 7th screening stage, in accordance with national referral criteria [16].

### Statistical analyses

Baseline characteristics were compared between children with and without ROP using *t*-tests for continuous variables and chi-squared tests for categorical variables. Prevalence rates were expressed per 100 individuals. The risk of developmental delay was evaluated by using multivariable logistic regression models to estimate odds ratios (ORs) with corresponding 95% confidence intervals (CIs). To account for potential confounding, both child and maternal covariates were included in the models. The child covariates comprised sex, birth weight, multiple birth status, visual impairment, major congenital anomalies, epilepsy, and hearing loss. The maternal covariates included gestational diabetes mellitus, pregestational diabetes mellitus, pregnancy-induced hypertension, chronic hypertension, depression, psychotic disorders, bipolar disorder, and the use of or dependence on substances such as tobacco, alcohol, or drugs. All statistical analyses were conducted using SAS software (version 9.4, SAS Institute, Cary, NC). The significance criterion was set as *p* < 0.05.

## Results

### Study cohort

**Fig 1:**
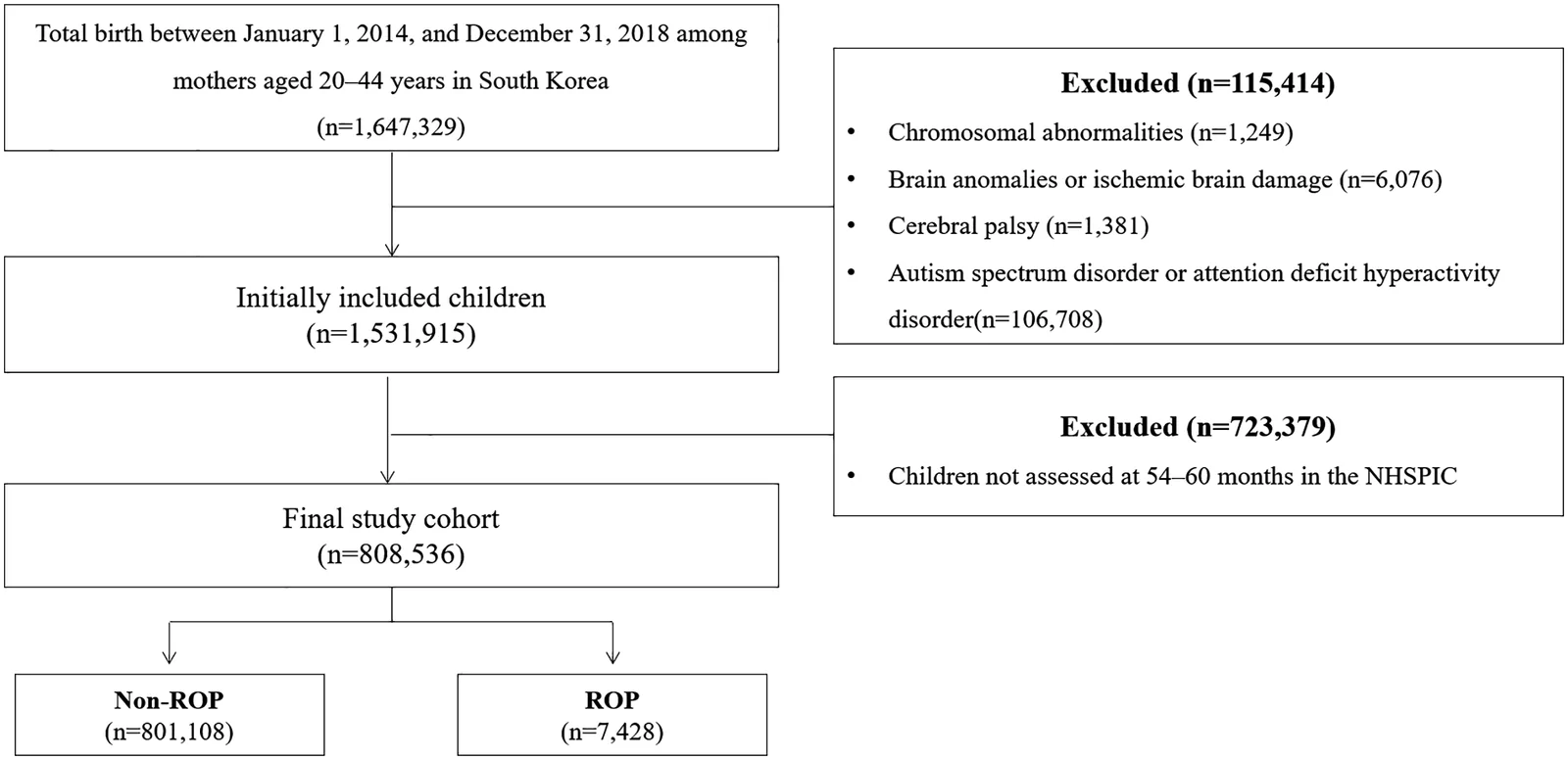
Flowchart of the study population. From 1,647,329 live births in South Korea during 2014–2018, after applying the study exclusion criteria, 808,536 children remained eligible for analysis. Among them, 7,428 were diagnosed with retinopathy of prematurity (ROP).

Between January 1, 2014 and December 31, 2018 there were 1,647,329 live births recorded among mothers aged 20–44 years in South Korea. After applying the study exclusion criteria, 1,531,915 children remained eligible. The final study cohort consisted of 808,536 children who participated in NHSPIC assessments at 54–60 months of age (Fig 1), including 7,428 (0.92%) diagnosed with ROP.

### Baseline characteristics of the study population

Table 1 summarizes the maternal and neonatal characteristics in the ROP and non-ROP groups. Children with ROP were more likely to be from multiple pregnancies and to present with hearing loss, epilepsy, subnormal VA, and major anomalies. The mean gestational age was significantly lower in the ROP group (33.1 weeks), which was accompanied by a lower mean birth weight than in the non-ROP group. The ROP group also exhibited significantly lower body weight, height, and VA at the 7th NHSPIC screening stage.

**Table 1:** Baseline characteristics of children with and without retinopathy of prematurity (ROP).

	Non-ROP	ROP	P-value
n	801108	7428	
%	99.08	0.92	
**Maternal factors**			
Age, year	32.2 ± 3.89	32.36 ± 4.12	<.0001
Age (≥ 35 years)	223860(27.94)	2623(35.31)	<.0001
Vaginal delivery	464947(58.04)	2237(30.12)	<.0001
Hypertension	38616(4.82)	1147(15.44)	<.0001
DM	11910(1.49)	318(4.28)	<.0001
PIH	96695(12.07)	1588(21.38)	<.0001
GDM	151511(18.91)	1522(20.49)	0.0006
Depression	74580(9.31)	824(11.09)	<.0001
Psychotic disorder	4163(0.52)	42(0.57)	0.5851
Bipolar disorder	4876(0.61)	54(0.73)	0.1923
Tobacco, alcohol or drug dependence	9161(1.14)	98(1.32)	0.1564
**Child factors**			
Multiple birth	25559(3.19)	2219(29.87)	<.0001
Sex, male	399451(49.86)	4035(54.32)	<.0001
Gestational age, weeks	35.98 ± 2.04	33.08 ± 2.46	<.0001
Birth weight	3.2 ± 0.46	2.23 ± 0.72	<.0001
Weight at 54–60 months	19.23 ± 3	18.83 ± 3.15	<.0001
Height at 54–60 months	108.97 ± 4.63	108.19 ± 4.54	<.0001
Visual acuity (VA) at 54–60 months (worse eye)	0.79 ± 0.22	0.76 ± 0.23	<.0001
Hearing Loss	8265(1.03)	598(8.05)	<.0001
Epilepsy	2232(0.28)	103(1.39)	<.0001
Major anomaly (excluding brain anomalies)	42303(5.28)	1255(16.9)	<.0001

Data are presented as mean±standard deviation for continuous variables and as number (%) for categorical variables. VA values are expressed using the decimal visual acuity scale.

PIH: pregnancy induced hypertension, DM: diabetes mellitus, GDM: gestational diabetes mellitus

Among maternal characteristics, higher maternal age (≥35 years at delivery) and cesarean section was more frequent in the ROP group. In addition, chronic maternal conditions such as hypertension and diabetes mellitus as well as pregnancy-related complications including gestational diabetes mellitus and pregnancy-induced hypertension were significantly more common among mothers in the ROP group. Regarding the maternal psychiatric condition, depressive disorder was more prevalent in the ROP group, while the prevalence rates of psychotic disorder, bipolar disorder, and substance dependence did not differ significantly between the ROP and non-ROP groups.

### Cross-sectional association between VA and developmental delay

Table 2 presents the adjusted odds ratios (aORs) for developmental delay according to the ROP status. The prevalence of developmental delay across all six K-DST domains was higher in children with ROP, although the association narrowly missed reaching statistical significance for the self-care domain. The observed aORs ranged from 1.346 for cognition (95% CI = 1.003–1.806) to 1.561 for gross motor skills (95% CI = 1.180–2.065).

**Table 2:** Risk of developmental delay according to ROP status.

Developmental domain	ROP	Event	PR (per 100)	Adjusted OR(95% CI)
Self-care	No	3161	0.39	1
	Yes	49	0.66	1.322(0.982-1.779)
Cognition	No	3128	0.39	1
	Yes	50	0.67	1.346(1.003-1.806)
Language	No	3466	0.43	1
	Yes	64	0.86	1.496(1.151-1.945)
Fine motor	No	3134	0.39	1
	Yes	55	0.74	1.447(1.091-1.918)
Gross motor	No	2812	0.35	1
	Yes	57	0.77	1.561(1.180-2.065)
Sociality	No	2767	0.35	1
	Yes	46	0.62	1.418(1.043-1.927)

ROP, retinopathy of prematurity; PR, prevalence rate; OR, odds ratio; CI, confidence interval

### Developmental outcomes stratified by ROP and VA

**Fig 2:**
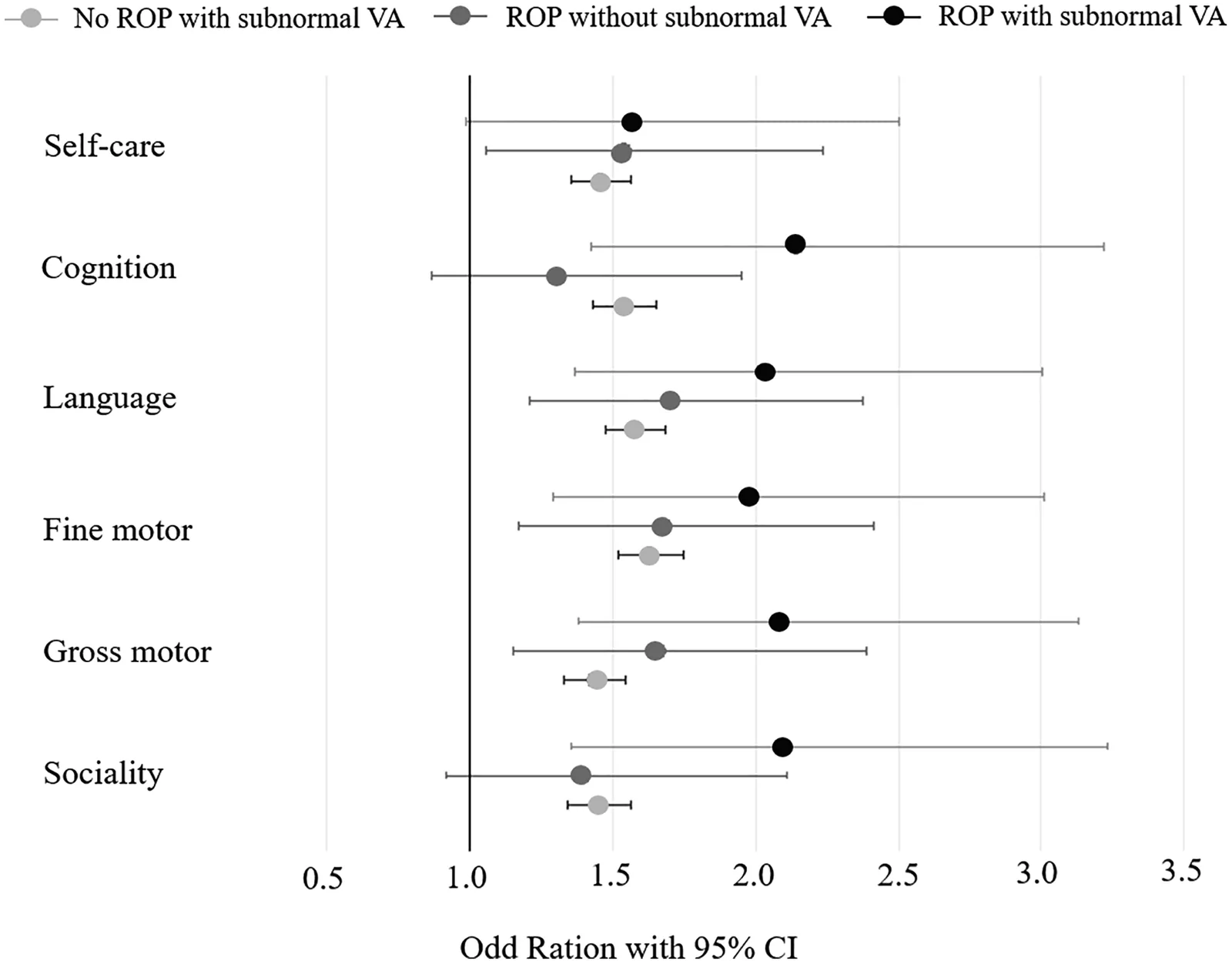
Adjusted odds ratios (aORs) with 95% confidence intervals (CIs) for developmental delay across six domains (cognition, self-care, language, fine motor skills, gross motor skills, and sociality), stratified by ROP and visual acuity (VA) status. Children were categorized into four groups: (1) neither ROP nor subnormal VA, (2) no ROP with subnormal VA, (3) ROP without subnormal VA, and (4) both ROP and subnormal VA. Developmental delays were consistently more likely in children with ROP, particularly those with concomitant subnormal VA.

To further separate the effect of ROP from that of subnormal VA, children were classified into four groups: neither ROP nor subnormal VA, no ROP with subnormal VA, ROP without subnormal VA, and both ROP and subnormal VA (Table 3). Across all domains, the prevalence rate of developmental delay increased progressively from those with neither ROP nor subnormal VA to those with both ROP and subnormal VA. Using those with neither ROP nor subnormal VA as the reference group, the risks of developmental delay across all six K-DST domains were significantly higher in those with no ROP with subnormal VA and in those with both ROP and subnormal VA (aORs = 1.436–2.143) (Fig 2). The risks in the development of self-care, language, fine motor skills, and gross motor skills were significantly elevated in those with ROP without subnormal VA (aORs = 1.540–1.698), while the associations but did not reach statistical significance in the cognition and sociality domains (Table 3).

**Table 3:** Risk of developmental delay stratified by ROP and subnormal visual acuity (VA) status.

Developmental domain	ROP / subnormal VA	N	event	PR (per 100)	Adjusted OR (95% CI)
Self-care	No / No	533721	1821	0.34	1
	No / Yes	267387	1340	0.5	1.459(1.359-1.566)
	Yes / No	4594	30	0.65	1.54(1.061-2.236)
	Yes / Yes	2834	19	0.67	1.573(0.990-2.501)
Cognition	No / No	533721	1759	0.33	1
	No / Yes	267387	1369	0.51	1.54(1.434-1.653)
	Yes / No	4594	25	0.54	1.304(0.869-1.955)
	Yes / Yes	2834	25	0.88	2.143(1.426-3.219)
Language	No / No	533721	1928	0.36	1
	No / Yes	267387	1538	0.58	1.579(1.476-1.689)
	Yes / No	4594	37	0.81	1.698(1.212-2.379)
	Yes / Yes	2834	27	0.95	2.029(1.371-3.001)
Fine motor	No / No	533721	1717	0.32	1
	No / Yes	267387	1417	0.53	1.632(1.521-1.752)
	Yes / No	4594	32	0.7	1.682(1.172-2.416)
	Yes / Yes	2834	23	0.81	1.973(1.292-3.013)
Gross motor	No / No	533721	1628	0.31	1
	No / Yes	267387	1184	0.44	1.436(1.332-1.548)
	Yes / No	4594	32	0.7	1.66(1.154-2.387)
	Yes / Yes	2834	25	0.88	2.08(1.382-3.131)
Sociality	No / No	533721	1602	0.3	1
	No / Yes	267387	1165	0.44	1.449(1.343-1.563)
	Yes / No	4594	24	0.52	1.394(0.921-2.11)
	Yes / Yes	2834	22	0.78	2.094(1.358-3.231)

No / No = ROP (-) / subnormal VA (-); No / Yes = ROP (-) / subnormal VA (+); Yes / No = ROP (+) / subnormal VA (-); Yes / Yes = ROP (+) / subnormal VA (+)

## Discussion

This study evaluated developmental screening outcomes at 54–60 months of age among children with and without a history of ROP using nationwide NHSPIC data. In this cohort of more than 800,000 children, a history of ROP was associated with higher odds of developmental delay across multiple K-DST domains after adjustment for available maternal and child covariates. In stratified analyses according to ROP and presenting VA status, children with both ROP and subnormal presenting VA showed the greatest odds of developmental delay. Elevated odds were also observed in several developmental domains among children with ROP but without subnormal presenting VA. These findings suggest that ROP history may help identify preschool children with developmental vulnerability, particularly when reduced functional visual status is also present.

ROP is a major cause of childhood blindness, but increasing evidence suggests that children with ROP may also be vulnerable to adverse neurodevelopmental outcomes [17,18]. Previous studies have reported associations between ROP and cognitive impairment, cerebral palsy, and behavioral problems; however, these relationships are difficult to disentangle from prematurity, low birth weight, oxygen exposure, neonatal illness severity, and visual impairment [19,20]. Our findings are consistent with this literature by showing that children with a history of ROP had higher odds of developmental screening abnormalities at 54–60 months, including those without subnormal presenting VA. These associations may reflect shared prematurity-related mechanisms rather than a direct effect of ROP itself. Retinal and cerebral development involve overlapping neurovascular pathways, and perinatal insults associated with ROP may also affect brain and visual pathway maturation [21]. Recent neuroimaging and histologic studies have revealed that preterm infants exhibit alterations in both the retinal cellular architecture and along the dorsal visual stream, with structural changes between and within cortical areas [22]. Sustained systemic inflammation during the perinatal period has also been implicated in both ROP and subsequent neurodevelopmental deficits [23]. In this context, the concept of visuopathy of prematurity (VOP) provides a useful framework in which ROP is viewed as one manifestation of broader neurovascular vulnerability involving the retina, choroid, visual pathways, and cerebral cortex [24].

These results are consistent with a growing body of evidence suggesting that ROP may be a clinical marker of prematurity-related neurovascular vulnerability rather than an isolated ocular disease. Systematic reviews and meta-analyses have reported associations between ROP and cognitive impairment or intellectual disability, cerebral palsy, and behavioral problems, with more severe ROP generally showing stronger associations with adverse neurodevelopmental outcomes. Our findings are in line with these reports by showing that children with a history of ROP, particularly those with subnormal presenting VA, had elevated odds of domain-specific developmental screening abnormalities in a large nationwide cohort. For example, children with both ROP and subnormal presenting VA showed elevated odds of fine motor and gross motor delays. These findings are clinically plausible, given the close relationship between visual function and motor development [25]. Previous research has also suggested that severe ROP may be associated with altered cerebellar and brainstem development and later neurodevelopmental deficits, including motor impairment [26].

The elevated odds of language and sociality delays in children with ROP and/or subnormal presenting VA are consistent with previous studies suggesting that reduced visual function may affect exploratory behavior, referent learning, social communication, and early cognitive development. These processes may influence the acquisition of language and social milestones [27–29]. In the self-care domain, children with both ROP and subnormal presenting VA showed numerically higher odds of delay, but the association was not statistically significant and should be interpreted cautiously. The overall pattern suggests that reduced functional visual status may be related to difficulties in adaptive skills that rely partly on visual observation and imitation.

In terms of clinical implications, our findings suggest that children with a history of ROP may benefit from developmental surveillance during the preschool period, particularly when subnormal presenting VA is also present. Early identification of developmental screening abnormalities in language, cognitive, and motor domains may provide opportunities for timely referral and intervention. In the stratified analysis, children with ROP but without subnormal presenting VA showed elevated odds of delay in self-care, language, fine motor, and gross motor domains. Although these findings may partly reflect residual confounding by prematurity-related factors, they suggest that ROP history may help identify children with broader developmental vulnerability beyond reduced presenting VA alone. Children with both ROP and subnormal presenting VA showed the greatest odds of developmental delay across several domains. Therefore, ROP history, especially when accompanied by reduced functional visual status, may serve as a practical clinical signal for integrated ophthalmic and developmental follow-up before school entry.

The association between subnormal presenting VA and developmental screening abnormalities also has important clinical implications. However, presenting VA does not identify the underlying cause of reduced vision or represent best-corrected VA after cycloplegic refraction. From a neurodevelopmental perspective, adequate visual input during early childhood is important for the maturation of cognitive, motor, and visuospatial functions [30]. Reduced functional visual status may limit visual exploration, visuomotor integration, and environmental learning, which are relevant to several developmental domains [29,31]. In preschool children, reduced presenting VA may result from potentially correctable causes such as uncorrected refractive error, as well as amblyopia, strabismus, or structural ocular disease [32]. Previous research has shown that uncorrected refractive errors negatively affect the cognitive, motor, and psychosocial development of children, while the timely implementation of correction using spectacles has been shown to improve both the quality of life and educational performance [33]. Therefore, identifying subnormal presenting VA may provide an opportunity for timely ophthalmologic evaluation and visual rehabilitation, while its association with developmental screening outcomes should be interpreted cautiously.

This study analyzed developmental screening outcomes at the 7th NHSPIC screening stage, corresponding to 54–60 months of age. This time point was selected because it provides a standardized preschool-age assessment that includes both presenting VA and K-DST developmental evaluation within the same screening period. Therefore, the results should be interpreted as a cross-sectional screening snapshot before school entry rather than as evidence of longitudinal developmental trajectories. Although earlier screening stages may be more relevant for detecting early developmental delay, VA assessment in younger children can be limited by cooperation and testability. Conversely, later developmental outcomes may be increasingly influenced by educational, environmental, and social factors. Thus, the 54–60-month screening stage provided a practical time point for evaluating the association between ROP history, presenting visual status, and preschool developmental screening outcomes within the national health screening system.

A major strength of this study is the use of a nationwide mother–child linked database that enabled evaluation of maternal, perinatal, ophthalmic, and developmental information within a large national health screening system. The large sample size and standardized NHSPIC protocol allowed us to characterize developmental screening outcomes across multiple K-DST domains among preschool children with and without a history of ROP. In addition, presenting VA and developmental status were assessed during the same screening period, providing a practical framework for examining the relationship between ROP history, functional visual status, and preschool developmental vulnerability.

Several limitations should be considered. First, residual confounding by prematurity-related factors remains the most important limitation. ROP is closely associated with gestational age, birth weight, oxygen exposure, neonatal illness severity, NICU course, and other neonatal morbidities. Although the analysis adjusted for available maternal and child covariates, detailed neonatal variables such as oxygen therapy, mechanical ventilation, bronchopulmonary dysplasia, sepsis, and intraventricular hemorrhage were unavailable. Therefore, the observed associations should not be interpreted as evidence that ROP independently causes developmental delay. Second, ROP was identified using the ICD-10 code H35.1, without information on stage, zone, laterality, timing, spontaneous regression, laser treatment, or anti-VEGF therapy. Thus, disease severity and dose–response relationships could not be evaluated. Third, presenting VA does not identify the cause of reduced vision or represent best-corrected VA after cycloplegic refraction. Conditions such as uncorrected refractive error, amblyopia, strabismus, structural ocular disease, and CVI could not be distinguished. Fourth, because the final cohort included only children who completed the 54–60-month screening, selection bias related to follow-up participation cannot be excluded. Fifth, the exclusion of children with previously coded ASD, ADHD, CP, and other major neurologic or developmental diagnoses may have removed clinically severe cases and limits generalizability to the full spectrum of neurodevelopmental impairment. Finally, developmental outcomes were assessed at a single preschool-age screening point and should be interpreted as cross-sectional screening findings rather than longitudinal developmental trajectories [34]. Future studies with detailed neonatal, ophthalmic, socioeconomic, and longitudinal developmental data are needed.

In conclusion, this nationwide health screening study found that children with a history of ROP had higher odds of developmental screening abnormalities at 54–60 months, particularly when subnormal presenting VA was also present. Because ROP is closely linked to prematurity-related neonatal vulnerability, these findings should be interpreted as indicating that ROP may serve as a clinical marker of developmental vulnerability rather than as an independent causal factor. Integrated ophthalmic and developmental surveillance may be useful for preschool children with a history of ROP, especially when reduced functional visual status is present.

## Supporting information

S1 Table Codes used to define the exclusion criteria and covariates. (DOCX)
