# Supplementary material for: Retinopathy of prematurity as a clinical marker of developmental vulnerability in preschool children: A nationwide Korean Health Screening study
Source: PLoS One. 2026 Sep 2;21(9):e0356773. doi: 10.1371/journal.pone.0356773 (PMC13537600; doi:10.1371/journal.pone.0356773)
Supplement: Supplementary file 1 [file pone.0356773.s001.docx]

**S1 Table.** Codes used to define the exclusion criteria and covariates

| **Categories** | **ICD-10 codes** |
| --- | --- |
| **Study population selection** | |
| Retinopathy of prematurity | H35.1 |
| Autism spectrum disorders | F84.0, F84.1, F84.5 |
| Unspecified speech or language developmental disorders | F80.9 |
| Unspecified developmental disorders | F89 |
| Attention-deficit/hyperactivity disorders | F90.0, F90.1 |
| Delayed developmental milestones | R62.0 |
| Brain injuries or congenital brain anomalies | periventricular leukomalacia (P91.2), neonatal cerebral ischemia (P91.0), hypoxic ischemic encephalopathy (P91.6), anoxic brain damage (G93.1), anencephaly (Q00), microcephaly (Q02), and other congenital malformations of the brain (Q04). |
| Cerebral palsy | G80 |
| Teratogenic/genetic syndromes, microdeletions, and chromosomal abnormalities | Q90-Q92, Q930-Q939, and Q95-Q99 |
| **Covariates** | |
| Hearing impairment | H90.3, H90.5, H91.9 |
| Epilepsy | G40 |
| Major congenital malformations |  |
| Nervous system | Q00-Q07 |
| Eye | Q100, Q104, Q106-Q109, Q11-Q12, Q130-Q134, Q136-Q139, and Q14-Q15 |
| Ear, face, and neck | Q16, Q176-Q178, Q183, and Q188 |
| Heart defects | Q2,; Q21, Q22, Q23, Q24, Q25,Q260, Q262-Q269 |
| Respiratory system | Q300, Q321-Q329, Q330, Q332-Q335, Q337-Q339, and Q34 |
| Oral clefts | Q35-Q37 |
| Digestive system | Q380, Q383-Q389, Q39; Q402-Q409, Q41, Q42, Q431-Q439, Q44, Q45, Q790 |
| Abdominal wall defects | Q792-Q793 and Q795 |
| Urinary system | Q60, Q611-Q619, Q620-Q626, Q628-Q629, Q630-Q632, Q634-Q639, Q64, and Q794 |
| Genital organs | Q50-Q51, Q520-Q522, Q524, Q526, Q528-Q529, and Q54-Q56 |
| Limb | Q660-Q661, Q679, Q681-Q682, Q686-Q689, and Q70-Q74 |
| Maternal conditions |  |
| Psychotic disorder | F20-F25, F29 |
| Bipolar disorder | F30-F31 |
| Depressive disorder | F32–33 |
| Substance related disorder | F10-19, Z71.4, Z71.5, Z71.6, Z72.0, Z72.1, and Z72.2 |
| Gestational diabetes mellitus (GDM) | O244, O249 |
| Pregnancy induced hypertension(PIH) | O12-15 |
| Diabetes Mellitus | E11-E14 |
| Chronic hypertension | I10-13, I15 |

**Abbreviations:** ICD-10, International Classification of Diseases 10^th^ revision
