# Supplementary material for: Retinopathy of prematurity as a clinical marker of developmental vulnerability in preschool children: A nationwide Korean Health Screening study
Source: PLoS One. 2026 Sep 2;21(9):e0356773. doi: 10.1371/journal.pone.0356773 (PMC13537600; doi:10.1371/journal.pone.0356773)
Supplement: Supplementary file 2 [file pone.0356773.s003.docx]

**Response to Reviewers**

**Retinopathy of Prematurity as a Clinical Marker of Developmental Vulnerability in Preschool Children: A Nationwide Korean Health Screening Study**

We are deeply grateful for reviewer’s sincere and valuable comments that have resulted in significant manuscript improvements. We hope that our responses satisfactorily address reviewer’s concerns.

**Reviewer response**

**Comment:**
Is VA in logMAR? Just indicate in details in the manuscript.

**Response:**
Thank you for this comment. Visual acuity was recorded using the decimal visual acuity scale, rather than the logMAR scale, in the National Health Screening Program for Infants and Children. Accordingly, all visual acuity values and referral thresholds reported in this study are expressed on the decimal scale, and no conversion to logMAR was performed. We have clarified the visual acuity scale in the Methods section and Table 1 as following:

Line 139-140
“VA was evaluated during the 6th (42–48 months), 7th (54–60 months), and 8th (66–71 months) screening stages of the NHSPIC and was recorded using the decimal visual acuity scale. This study used VA measurements from the 7th screening stage, corresponding to the same screening period as the developmental assessment.”

Line 142-143

According to the NHSPIC vision screening guidelines, children are referred for further ophthalmologic evaluation if their presenting decimal VA is <0.5 at the 5th screening stage or <0.6 or <0.63, depending on the optotype chart format, at the 6th and 7th screening stages.

Line 148-179

In this study, subnormal presenting VA was defined as decimal VA <0.6 or <0.63, depending on the chart format, in at least one eye at the 7th screening stage, in accordance with the national referral criteria [16].

Table 1
footnote: “VA values are expressed using the decimal visual acuity scale.”
